# Determinants of condom breakage among female sex workers in Karnataka, India

**DOI:** 10.1186/1471-2458-11-S6-S14

**Published:** 2011-12-29

**Authors:** Janet Bradley, S Rajaram, Michel Alary, Shajy Isac, Reynold Washington, Stephen Moses, BM Ramesh

**Affiliations:** 1 CHARME-India Project, Bangalore; India KHPT office, IT Park 5th floor, #1-4 Rajajinagar Industrial Area, Behind KSSIDC Admin Office, Rajajinagar, Bangalore 560 044, India; 2 Centre hospitalier affilié universitaire de Québec, Canada; Unité de recherche en santé des populations, Centre de recherche du CHA de Québec, Hôpital du Saint-Sacrement, 1050 chemin Ste-Foy, Québec G1S 4L8, Canada; 3 Karnataka Health Promotion Trust, Bangalore, India; IT Park 5th floor, #1-4 Rajajinagar Industrial Area, Behind KSSIDC Admin Office, Rajajinagar, Bangalore 560 044, India; 4 Centre for Global Public Health, Faculty of Medicine, University of Manitoba, 771 Mc Dermot Avenue, Medical Rehabilitation Building, Room R070, Winnipeg, Manitoba R3E 0T6, Canada

## Abstract

**Background:**

Condoms are effective in preventing the transmission of HIV and other sexually transmitted infections, when properly used. However, recent data from surveys of female sex workers (FSWs) in Karnataka in south India, suggest that condom breakage rates may be quite high. It is important therefore to quantify condom breakage rates, and examine what factors might precipitate condom breakage, so that programmers can identify those at risk, and develop appropriate interventions.

**Methods:**

We explored determinants of reported condom breakage in the previous month among 1,928 female sex workers in four districts of Karnataka using data from cross-sectional surveys undertaken from July 2008 to February 2009. Using stepwise multivariate logistic regression, we examined the possible determinants of condom breakage, controlling for several independent variables including the district and client load.

**Results:**

Overall, 11.4% of FSWs reported at least one condom break in the previous month. FSWs were much more likely to report breakage if under 20 years of age (AOR 3.43, p = 0.005); if divorced/ separated/widowed (AOR 1.52, p = 0.012); if they were regular alcohol users (AOR 1.63, p = 0.005); if they mostly entertained clients in lodges/rented rooms (AOR 2.99, p = 0.029) or brothels (AOR 4.77, p = 0.003), compared to street based sex workers; if they had ever had anal sex (AOR 2.03, p = 0.006); if the sex worker herself (as opposed to the client) applied the condom at last use (AOR 1.90, p < 0.001); if they were inconsistent condom users (AOR 2.77, p < 0.001); and if they had never seen a condom demonstration (AOR 2.37, p < 0.001).

**Conclusions:**

The reported incidence of condom breakage was high in this study, and this is a major concern for HIV/STI prevention programs, for which condom use is a key prevention tool. Younger and more marginalized female sex workers were most vulnerable to condom breakage. Special effort is therefore required to seek out such women and to provide information and skills on correct condom use. More research is also needed on what specific situational parameters might be important in predisposing women to condom breakage.

## Introduction

Condom use is a key strategy for preventing sexually transmitted infections, including HIV [1,2]. However, condoms are only effective in preventing infection if they do not break or slip off during intercourse, and if they are correctly applied before initial penetration [3-7]. Published data show that condoms break approximately 1-13% of the time, depending on the population [8]. Data from Africa and India generally show much higher rates of breakage. In a study of female sex workers (FSWs) in Benin in 2005, Mukenge-Tshibaka et al. [9] reported that 33% had experienced a breakage in the previous 2 days. Data from female sex workers in four southern states of India are available from face-to-face interviews (FTFI) in cross-sectional studies termed integrated biological and behavioural assessments (IBBAs). The percentage of FSWs reporting condom breakage at least once in the last month ranged from 5% in Thane, Maharashtra (MH) to 55% in Vizag, Andhra Pradesh (AP), with rates generally very high in districts in AP (ranging from 29% to 55%), and low in districts in Tamil Nadu (ranging from 2% in Chennai to 14% in Dharmapuri) [10].

The objective of this exploratory study was to compare, in a large sample of female sex workers in four districts of Karnataka south India, the personal characteristics of those who reported a condom breakage in the previous month with those who did not. We examined aspects such as socio-demographic background, usual place of solicitation and place of sex, other aspects of sex work (e.g. practice of anal sex), client load, condom use, HIV knowledge, other risk factors such as alcohol use and history of violence, and exposure to programmatic interventions. The findings are important for determining if some women are more susceptible to breakage than others and thus for developing targeted programme interventions for those most at risk.

## Methods

As part of the evaluation of the Indian *Avahan* AIDS intervention, two rounds of IBBAs were undertaken in several urban areas of four south India districts of Karnataka (Belgaum, Bellary, Bangalore and Shimoga), with the Round 2 surveys, that we focus on here, done between July 2008 and February 2009 [11]. The studies included a face-to-face interview and taking of blood samples for STI and HIV testing with a total of 1,975 female sex workers, of whom 1,928 reported they had used condoms in the last month. One of the questions asked in the survey was whether the respondent experienced a condom breakage during use in the previous month. The respondents were selected randomly using time-location cluster sampling, based on a previously developed map of urban sex work sites that served as a sampling framework [11]. The data were merged and appropriate weights added to account for a complex sampling design. After univariate analysis, some variables, where the odds ratios were similar for different categories, were re-categorized for simplicity, and we included in the first model, only those variables that were significantly associated with breakage in univariate analysis (p < 0.05). We used stepwise logistic regression in a series of model iterations to determine the factors most associated with condom breakage, controlling also for both district and client load.

## Results

Of 1,928 FSWs interviewed, 220 (11.4%) reported that they had experienced a breakage in the month prior to the survey. On univariate analysis, compared to the entire sample, 17.1% of those who were found to be HIV positive reported a break (OR 1.78, *p*=0.008), as did 20.7% of those with chlamydial infection (OR 2.17, *p*=0.001), and 30.0% of those with gonorrhoea (OR 3.54, *p*=0.001). Twenty-two percent of the women who reported a breakage were in fact HIV positive, compared to 14% of those who did not report a breakage.

Table 1 shows factors in univariate analysis associated with condom breakage; some factors were not associated with breakage and so are not included in Table 1 (literacy, being in debt, age at first sex, how much the last client paid for sex, heard of STIs, heard of HIV, felt at risk of HIV, ever taken an HIV test). Breakage was least likely to be reported in Shimoga (6.1%) and most likely in urban Bangalore (16.7%). Women with more clients were, as expected, more likely to have had a breakage in the previous month, with those having more than twenty per week almost twice as likely to report breakage than those only having 1-4 clients. Some demographic factors and sex work practices appeared to be associated with reporting of breakages. Young women under the age of 20 years were much more likely to report breakage than older women, as were women who were divorced, separated or widowed, those new to sex work, and those who had also practiced sex work in other towns. Women who reported that they had ever had anal sex and those who reported violence, or had been arrested, were twice as likely to say that they had experienced a condom break. Those women who entertained their clients at home reported breakage less than those working elsewhere; women working in brothels reported breakage four times more than women entertaining at home. Alcohol use appeared to be an important factor; women who said they drink alcohol were 2-3 times more likely to report a condom break in the month before the survey.

**Table 1:** Univariate analysis of factors associated with condom breakage

Factor	Response categories	% broke in last month	Odds ratio	95% confidence intervals	*p* value
District	Shimoga Belgaum Bellary Bangalore	6.09 12.65 9.15 16.70	Ref 2.23 1.55 3.09	1.28-3.90 0.81-2.99 1.78-5.36	0.005 0.188 <0.001

Number of clients in typical week	1-4 5-9 10-19 20+	9.59 8.73 10.99 16.30	Ref 0.90 1.16 1.83	0.55-1.49 0.75-1.80 1.13-2.97	0.684 0.497 0.014

**Demographic factors**					

Age	40+ 35-39 30-34 20-24 <20	9.12 10.17 13.00 12.78 30.56	Ref 1.12 1.49 1.46 4.39	0.71-1.79 0.96-2.31 0.91-2.35 2.44-7.89	0.608 0.075 0.116 0.000

Marital status	Devadasi (traditional FSW) Divorced/separated/widowed Never married Married	4.84 13.58 12.03 10.57	Ref 3.09 2.69 2.32	1.36-7.03 1.07-6.73 1.04-5.21	0.007 0.035 0.041

**Sex work and personal risk factors**					

Duration of sex work in this district	>5 years 1-4 years < 1 year	10.20 11.29 18.23	Ref 1.12 1.96	0.78-1.61 1.09-3.53	0.538 0.024

Ever practiced sex in a different place	No Yes	10.16 20.93	Ref 2.34	1.54-3.55	<0.001

Ever had anal sex	No Yes	10.17 19.80	Ref 2.18	1.39-3.42	0.001

Been forced to have sex in the last year	No Yes	10.53 20.08	Ref 2.13	1.37-3.32	0.001

Ever been arrested	No Yes	10.15 21.50	Ref 2.42	1.54-3.81	<0.001

Main place of entertaining clients	Public places Home Rented room/lodge Brothels	6.42 7.54 13.80 21.95	Ref 1.19 2.32 4.10	0.51-2.77 1.02-5.32 1.67-10.08	0.686 0.046 0.002

Drink alcohol	Never Occasionally Regularly	8.27 21.00 17.03	Ref 2.95 2.28	1.71-5.09 1.63-3.17	<0.001 <0.001

**Condom use**					

Always used condoms in the last 30 days	Yes No	9.08 23.77	Ref 3.12	2.24-4.36	0.001

Where last condom was obtained	Peer educator/health facility Client Other	10.36 16.17 20.93	Ref 1.66 2.29	1.08-2.57 1.42-3.69	0.020 0.001

Last time condom was put on by?	Client Respondent	7.65 16.30	Ref 2.35	1.70-3.26	<0.001

**Programme exposure**					

Registered with sex worker CBO	Yes No	10.17 21.65	Ref 2.44	1.66-3.59	<0.001

Ever given condom by a peer educator	Yes No	10.28 21.43	Ref 2.38	1.62-3.50	<0.001

Ever seen a condom demonstration	Yes No	10.14 23.91	Ref 2.79	1.94-4.00	<0.001

Aspects of condom use were also important. Those women who received their last condom from a peer educator (i.e. a government condom) were much less likely to report a condom break than those who received them elsewhere. Consistency of condom use was important: those who had not used condoms every time in the previous month were three times more likely to report that one had broken. Interestingly, women who said they themselves had been the ones to apply the last male condom they had used were more than twice as likely to report a breakage as when the client applied the condom. Programme exposure variables were also included in the analysis. Respondents who said that they were registered with a sex worker community-based organization (CBO), were two and a half times less likely to report breakage than those women who were not part of a CBO. Similarly if they had ever received condoms from a peer educator and if they had seen a condom demonstration, they were two to three times less likely to report breakage than those without this type of exposure.

In the logistic regression model (Table 2), we found that respondents under 20 years of age were more than three times more likely than women aged 20 or older to have reported a condom breakage in the previous month (AOR 3.42, 95% CI 1.89-6.23, p<0.001); divorced, separated or widowed women more likely to report breakage than unmarried or currently married women (AOR 1.52, 95% CI 1.10-2.10, p=0.012); users of alcohol had a higher risk if reporting breakage (AOR 1.61, 95% CI 1.16-2.28 p=0.005); those who primarily entertained clients in lodges/rented rooms (AOR 2.99, 95% CI 1.12-8.01. p=0.029) or brothels (AOR 4.78, 95% CI 1.69-13.48, p=0.003) were more likely to report breakage rather than those who primarily entertained their clients in public places. Those women who reported that they had ever had anal sex were twice more likely than others to report breakage (AOR 2.03, 95% CI 1.23-3.36, p=0.006). Those sex workers who reported that they had been the one to put the condom during the last time one was used were more likely to report breakage (AOR 1.90, 95% CI 1.35-2.68, p=<0.001) and if the sex workers reported inconsistent (less than 100%) condom use in the last month, they were almost three times more likely to report breakage than consistent condom users (AOR 2.77, 95% CI 1.87-4.11, p<0.001). Those who had never seen a condom demonstration were more than twice more likely to report breakage in the last month than those who had seen a demonstration (AOR 2.37, 95% CI 1.65-3.40, p<0.001). Other variables associated with sex practices and with other aspects of programme exposure were not associated with breakage in the multivariate regression model.

**Table 2:** Logistic regression analysis of factors associated with condom breakage

Factor	Response categories	Adjusted odds ratio	95% confidence intervals	*p* value
District	Shimoga Belgaum Bellary Bangalore	Ref 1.47 1.20 2.26	0.80-2.71 0.65-2.24 1.30-3.91	0.213 0.55 1 0.004

Number of clients in typical week	1-4 5-9 10-19 20+	Ref 0.84 1.18 1.37	0.50-1.41 0.70-1.97 0.77-2.43	0.50 4 0.54 1 0.273

**Demographic factors**				

Age	20 and above <20	Ref 3.43	1.89-6.23	<0.001

Marital status	Never married/married/devadasi Divorced/separated/widowed	Ref 1.52	1.10-2.10	0.012

**Sex work and personal risk factors**				

Ever had anal sex	No Yes	Ref 2.03	1.23-3.36	0.006

Main place of entertaining clients	Public places Home Rented room/lodge Brothels	Ref 1.9 2.99 4.78	1.35-2.68 1.12-8.01 1.69-13.48	0.196 0.029 0.003

Drink alcohol	Never Ever	Ref 1.63	1.16-2.28	0.005

**Condom use**				

Always used condoms in the last 30 days	Yes No	Ref 2.77	1.87-4.11	<0.001

Last time condom was put on by?	Client Respondent	Ref 1.90	1.35-2.68	<0.001

**Programme exposure**				

Ever seen a condom demonstration	Yes No	Ref 2.37	1.65-3.40	<0.001

## Conclusions and discussion

There have been many studies that have examined factors associated with condom breakage, though they often only evaluate aspects of the sex act where a break occurred, rather than looking at over-arching population variables. Furthermore, most studies have been done in developed countries and in non-commercial sex settings, and with a limited number of subjects. A limitation of this study is that the IBBA data do not give us specific details of the sex act in which the condom breaks, and we can only examine personal factors. A further limitation of this study is that we asked only about whether condom breakage had occurred in the past month, not the number of times that breakage occurred as a proportion of all sex acts (i.e. the frequency of condom breakage). More research is needed on the frequency of breakage to better assess the potential impact of condom breakage on the HIV epidemic among FSWs in the context of high condom use levels. However, this study is a useful complement to the literature because it examines, in a large sample of female sex workers with many partners, the background characteristics and general sexual practices that might predispose them to condom breakage, so that programme planners can know *who* to target with informational material.

Not having witnessed a condom demonstration and inconsistency of condom use were key determinants of reporting breakage in this study, as has been reported elsewhere [12,13]. The women in this study who reported a breakage were more likely to be alcohol users, also reported in other studies [14,15]. There have not been, to our knowledge, any studies of condom breakage that compare women who have vaginal and anal sex, although a study of heterosexual and gay men in Australia [16] found that gay men had higher rates of breakage. In this study, we found that women who practised anal sex were twice as likely to report breakages as those women who did not practice anal sex. Sex workers in Karnataka are being taught how to apply a condom on a man’s penis, yet those who reported this also reported more breakage, also found in another study in the United States [17]. Not observed in other studies, but likely correlated with inexperience, was the fact that young women under 20 years were over three times more likely to have reported a condom breakage than older women. In addition, these young women are likely to be found in brothels, where there was almost a 5-fold risk of breakage, than among women who entertained their clients in public places. This is an unexpected result, as we had believed that breakage was more likely to occur in less structured situations, such as having sex standing up, furtively or hurriedly, or in the dark [18]. Neither of these factors (age and brothel work) has been associated with breakage in other studies, but this is possibly because we had a large randomly sampled group in our study, whereas other studies may have focused on more homogeneous groups of respondents. These findings are therefore important for programme planning in the Indian context. Other factors, found to be important in other sex worker studies in Thailand and China [14,19], such as a history of violence were important in the univariate analysis but not significant when controlling for other factors in the regression analysis.

Accessing young girls in lodges, and especially brothels, is particularly difficult in India, as many are trafficked and under the legal age for sex [20,21]. However, there is clearly a need to continue to try to reach them, and once reached, to conduct condom demonstrations and talk about alcohol use, anal sex, and how to reduce condom breakage and disease transmission. Finally, more research is needed in this population around the details of practices in specific sexual encounters. As with many aspects of HIV transmission probability, we find that the youngest women, those who engage in risky activities such as inconsistent condom use, alcohol use and anal sex and those least exposed to HIV programmes, that have condom demonstrations as part of the programme, are also the most vulnerable to condom breakage. An important direction for research in this population now is a comprehensive study that can evaluate the relative contributions of personal versus circumstantial factors such as condom fit, lubricant use, multiple condom use, lack of experience, furtive sex, and rough sex act that have been found to be important in other studies [9,14,22-28]. For now, the sex worker programmes need to make a special effort to seek out women who appear to be more susceptible to breakage than others, and provide information and skills to help them reduce their risk for HIV infection.

## Competing interests

The authors declare that they have no competing interests.

## Authors’ contributions

JB conceived of the study, supervised the analysis and wrote the manuscript; SR conducted the data analysis and commented on the manuscript; MA, SI, SM and BMR provided support for data analysis; and SI, RW and BMR designed the original studies from which the data were extracted. MA, RW and SM provided constructive feedback on the manuscript.
